# Relationship between aging population, birth rate and disposable income per capita in the context of COVID-19

**DOI:** 10.1371/journal.pone.0289781

**Published:** 2023-08-09

**Authors:** Guangli Yang, Liangchen Zhang

**Affiliations:** School of Accountancy, Guangzhou Xinhua University, Guangzhou, China; Griffith University, AUSTRALIA

## Abstract

The outbreak of the COVID-19 in early 2020 and the recurring epidemic in later years have disturbed China’s economy. Moreover, China’s demographic dividend has been disappearing due to its fastest aging population and declining birth rate. The birth rates in eastern provinces of China are much lower than those of the western provinces. Considering the impacts of the COVID-19 and aging population, this paper focused on the relationship between birth rate and the disposable income and tried to find effective measures to raise China’s birth rate. We discovered through regression analysis that the link between per capita disposable income and birth rate is initially "reverse J" and later "inverted J", indicating that per capita disposable income will influence the birth rate. Women’s employment rate and educational level are negatively correlated with the birth rate. To raise the fertility rate in China, it is necessary to increase the marriage rate and the willingness to have children by raising the per capita disposable income and introducing effective tax relief policies.

## 1. Introduction

The outbreak of the COVID-19 in China in early 2020 hindered China’s economic development and there are sporadic reports to date of COVID-19 recurrence. [1]. In addition, the number of people aged 60 years and older has been increasing in recent years, and the pace of population ageing is much faster than in the past. What’s more, China has seen a decline in birth rates [2]. Therefore, how to improve the birth rate and promote economic development become an urgent problem to China. In this study, birth rate is the number of live birth thousand persons, and disposable income per capita refers to the amount of money that a population has left after taxes have been paid. Tax relief refers to preferential tax policies.

According to the 7th census statistics, China’s total fertility rate in 2020 stood at 1.3, which was below the international birth alert line. "Chinese population was older and consisted of fewer children". The declining newborn birth rate, increasing mortality rate and aging population will hinder China’s economic development. To address this problem, the Political Bureau meeting of the CPC Central Committee proposed to implement a three-child policy with supportive measures to increase the birth rate and promote a balanced population [3]. The Three-child policy, whereby a couple can have three children, is a family planning policy in China. This study believes tax relief should be one of the important supportive measures. Personal income tax has the greatest impact on residents’ life. Increasing deductions or tax exemptions for couples with children can effectively increase their disposable income and raise their willingness to have more children. In this way, the three-child policy is better supported. This study explores the relationship between birth rate and disposable income per capita. To provide a theoretical basis for empirical research, the theories about birth rate and the disposable income per capita are introduced separately in the second part.

By analyzing the population aged over 60 and under 14 in China, Chen [4] finds that the distribution of China’s population has changed significantly over the past decades. More policies and measures need to be adopted to encourage people to have more kids. Jing argues that to ensure the smooth implementation of three-child policy, we need to offering more help to parents [5]. Tax reform should be one of the priorities. By analyzing the relationship between policies, socializing the costs and birth rate, Xu et al. found that family friendly policies can improve the willingness of families to have children and can increase the birth rate [6]. Hong and Zhu found that the intention to have three children was related to women’s education, income, and employment [7]. Stay-at-home mothers with lower levels of education were more likely to have three children. Moreover, childbearing has been considered the leading role of women in traditional gender role concept, where most married women crave motherhood to live up to cultural norms. According to Jiang, men and women with traditional gender role beliefs could have higher fertility desire, and those with modern gender role beliefs tend to have lower fertility [8].

According to Chia and Li, increasing income tax relief for children’s educational expenses and adding deductions for childbirth, childcare, and parenting special children can reduce the financial strain of childbearing families, increase disposable income, and raise the birth rate [9]. According to Mu, the fertility intentions are influenced by various factors, such as disposable household income and childbirth cost [10]. Families are especially cost constrained when their children are very young. This decreases the fertility rate in China. Ma and Guo found that higher educational attainment and worse the economic status will lower the fertility intentions of women working full time [11]. The government need to increase the family income and reduce the childbirth cost to promote fertility rate. Zhong and Wang found that there is an "inverse J-shaped" pattern between GDP per capita and fertility, but only when a certain level of economic development is attained [12]. For countries with effective family policies and high gender equality, the promotion of fertility by economic development is more obvious.

Peng and Cheng empirically studied the relationship between fertility and personal income tax and found that the tax value of the personal exemption would increase the general fertility rate [13]. Liu and Sun found that higher childbirth costs will reduce people’s willingness to have children, hence lowering the number of children in the future [14]. Lan, Wu and Yu suggested that China should implement tax relief policies, such as deducting childbirth expenses on personal income tax and providing financial assistance to low-income families with children, so as to reduce or externalize childbirth costs to encourage childbirth [15].

In summary, the improvement of the birth rate, which is an important topic in China, has gained much attention from researchers. The birth rate is closely related to the residents’ willingness to have children, which is influenced by a variety factors, such as household disposable income, childbirth costs, gender of the newborn, women’s educational level, employment, etc. At present, most studies regard the birth rate and per capita disposable income as separate subjects. There hasn’t been much research done on their relationship. As fast aging population in China and the low birth rate can be affected by the COVID-19, this paper explores the relationship between birth rate and per capita disposable income, as well as the per capita disposable income and birth rate in the central, western, eastern and northeastern regions. What’s more, this study has proposed several measures to improve the birth rate, including reducing costs of housing, education, and medical care, increasing the marriage rate and willingness to have children, and introducing preferential policies for childbirth. China’s three north-eastern provinces—Heilongjiang, Jilin and Liaoning, among others, need to be paid special attention to stabilize the birth rate.

## 2. Fertility and disposable income per capita correlation theory

### 2.1 The overall birth rate shows a downward trend

**Fig 1:**
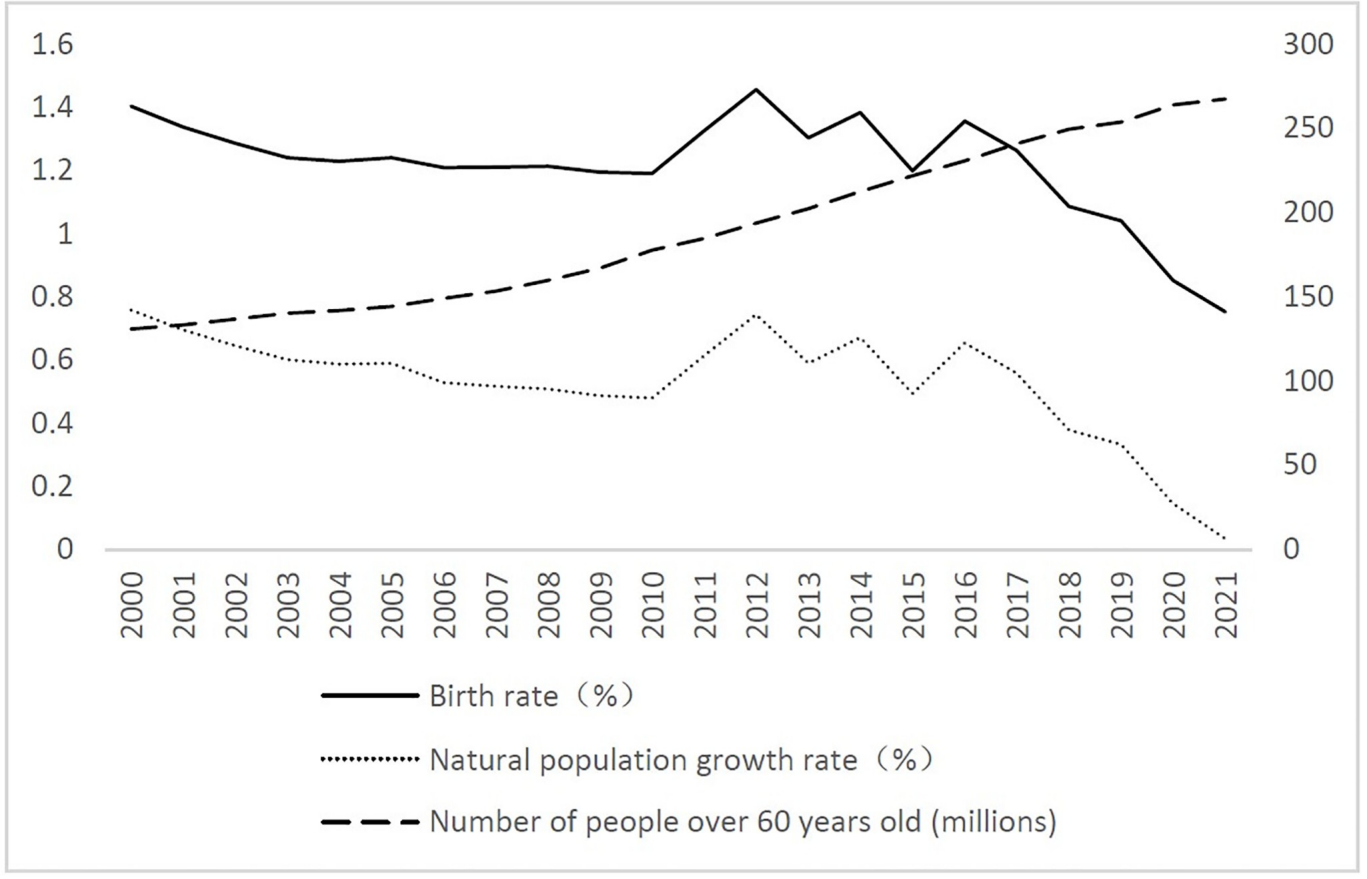
China’s birth rate, natural population growth rate and the degree of aging from 2000 to 2021.

According to the data of China National Bureau of Statistics, the growth rate of Chinese population is low in the past 10 years. From 2000 to 2010, the birth rate keeps falling (as shown in Fig 1). During this period, family planning policies were implemented. From 2010 to 2016, during which the two-child policy was implemented, the birth rate rebounded slightly. However, after 2017, the birth rate began to decline significantly. For example, the birth rate in 2020 was 0.852% and it fell to 0.752% in 2021, lower than 1%. The population aged 60 years and over has been rising significantly since 2008, from over 200 million in 2013 to 267 million in 2021. Population aging is caused by both the increase in life expectancy and the decrease in fertility rate. Economic growth and an improving quality of life have positive impacts on the aging population. Influenced by the two factors of decreasing birth rate and increasing aging population, the natural growth rate shows the same decreasing trend as the birth rate.

Du and Lin pointed that with China’s economic development, the gap between urban and rural areas is getting bigger, and residents’ living cost is increasing, especially housing and childbirth [16]. With a higher living pressure, residents’ fertility has also undergone a major change. Yu found that in the past, "parents raised children in the hope that when they grew up, they would take care of the old parents". However, such idea has gradually faded [17]. Nowadays, no one wants their children to lose at the starting line, and as a result, the education and training for their children is increasing at a higher level. Zhang, Zeng and Wu suggested that the demographic imbalance and the change of fertility concept caused the decline of labor supply, the rise of labor cost, and the gradual weakening of the demographic dividend [18]. This has a significant impact on the long-term development of economy. Therefore, it is very important to study how to improve the birth rate in China.

### 2.2 Relationship between disposable income per capita and birth rate

According to Li, Yang and Miao, since China began its reform and opening up, the eastern region has experienced rapid economic growth due to its geographical location and policy support [19]. Although central and western regions have also followed the policy to develop economies, they are lagging behind the eastern region and the income gap between coastal and western regions is huge. Economists attribute the difference partly to the location of most technology and finance companies in eastern areas. According to the data in Table 1, all the regions have seen a growing trend of per capita disposable income for both urban and rural residents. Compared to other parts of China, the eastern region has the highest income while the western region has the lowest income. As shown in Table 1, the ratio of per capita disposable income in the east to that in west regions stays at around 1.4. This ratio slightly decreased from 2013 to 2016, but rebounded from 2016 to 2020. The east-west imbalance becomes severe. Similarly, the east-west disposable income ratio declined in 2015 but climbed year after year following 2015. This indicates that the gap between the two regions continues to widen. The unbalanced economic development in the east and west has also resulted in disparities in birth rates. The eastern region had much lower birth rates relative to the western region. For example, the birth rate of Heilongjiang province dropped below 1% in 2001 and kept declining since then. In 2021, its birth rate declined to as low as 0.359%, making it the only province with a birth rate below 0.4%. In comparison, Yunnan and Guizhou both maintained birth rates above 1.2% in 2021. However, the birth rates in Beijing and Shanghai in the same year were 0.635% and 0.467%, respectively.

**Table 1:** Per capita disposable income of urban residents in different regions Unit: Yuan.

Group	Eastern Region	Central Region	Western Region	Northeast Region	Eastern/Western Region
2013	31152.4	22664.7	22362.8	23507.2	1.3930
2014	33905.4	24733.3	24390.6	25578.9	1.3901
2015	36691.3	26809.6	26473.1	27399.6	1.3860
2016	39651.0	28879.3	28609.7	29045.1	1.3859
2017	42989.8	31293.8	30986.9	30959.5	1.3874
2018	46432.6	33803.2	33388.6	32993.7	1.3907
2019	50145.4	36607.5	36040.6	35130.3	1.3914
2020	52027.1	37658.2	37548.1	35700.1	1.3856

Data source: China Statistical Yearbook (2020–2021).

**Fig 2:**
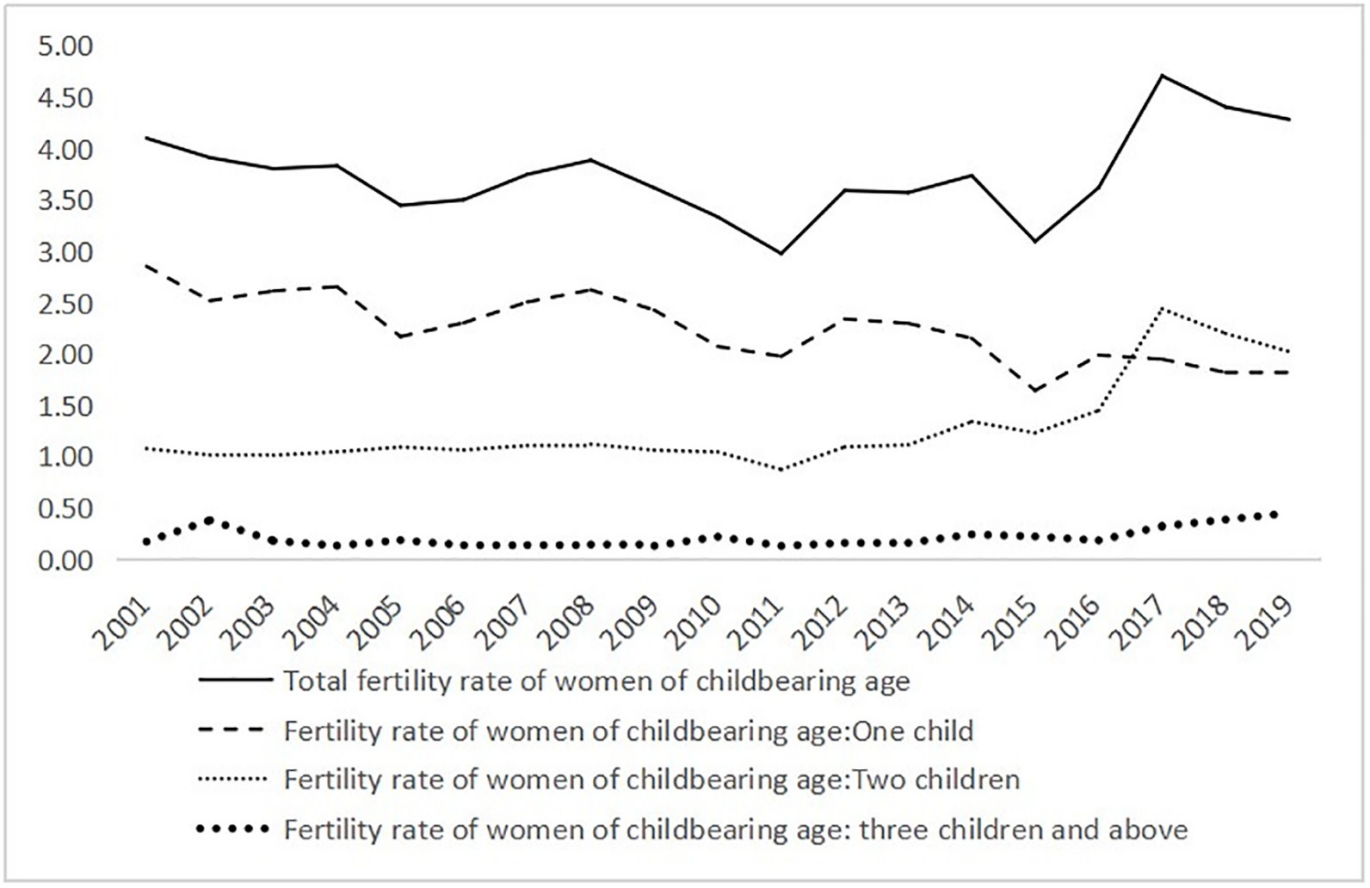
Fertility rate of women in the reproductive age range.

Fig 2 depicts the overall fertility rates of women in the reproductive age range who gave birth to one, two or three children. The information comes from the National Bureau of Statistics. On January 1, 2016, China further relaxed its family planning policy and adopted the universal two-child policy to actively address the country’s aging trend. As a result, the total fertility rate of women in the reproductive age range (15–49 years) rose significantly in 2016 and reached a record high of 4.7 in 2017. The fertility rate decreased then but was still above 4.2 in 2018 and 2019. The one-child fertility rate fell from 2.85 in 2001 to 1.81 in 2019, with a decrease of 36.5%, but the second-child fertility rate was relatively stable at around 1, with a small dip in 2011 and an increase from 2012. The second-child fertility rate rose the most to 2.44 in 2017. This indicates that the implementation of the two-child policy has a catalytic effect on the increase of fertility. The third-child fertility rate remained relatively stable, between 0.1 and 0.5, with an upward trend after 2017. Since 2021, the government has laid out a new three-child policy that permits couples to have a third child and provides incentives for them to do so. To increase the fertility rate, China could fully liberalize the birth policy.

**Fig 3:**
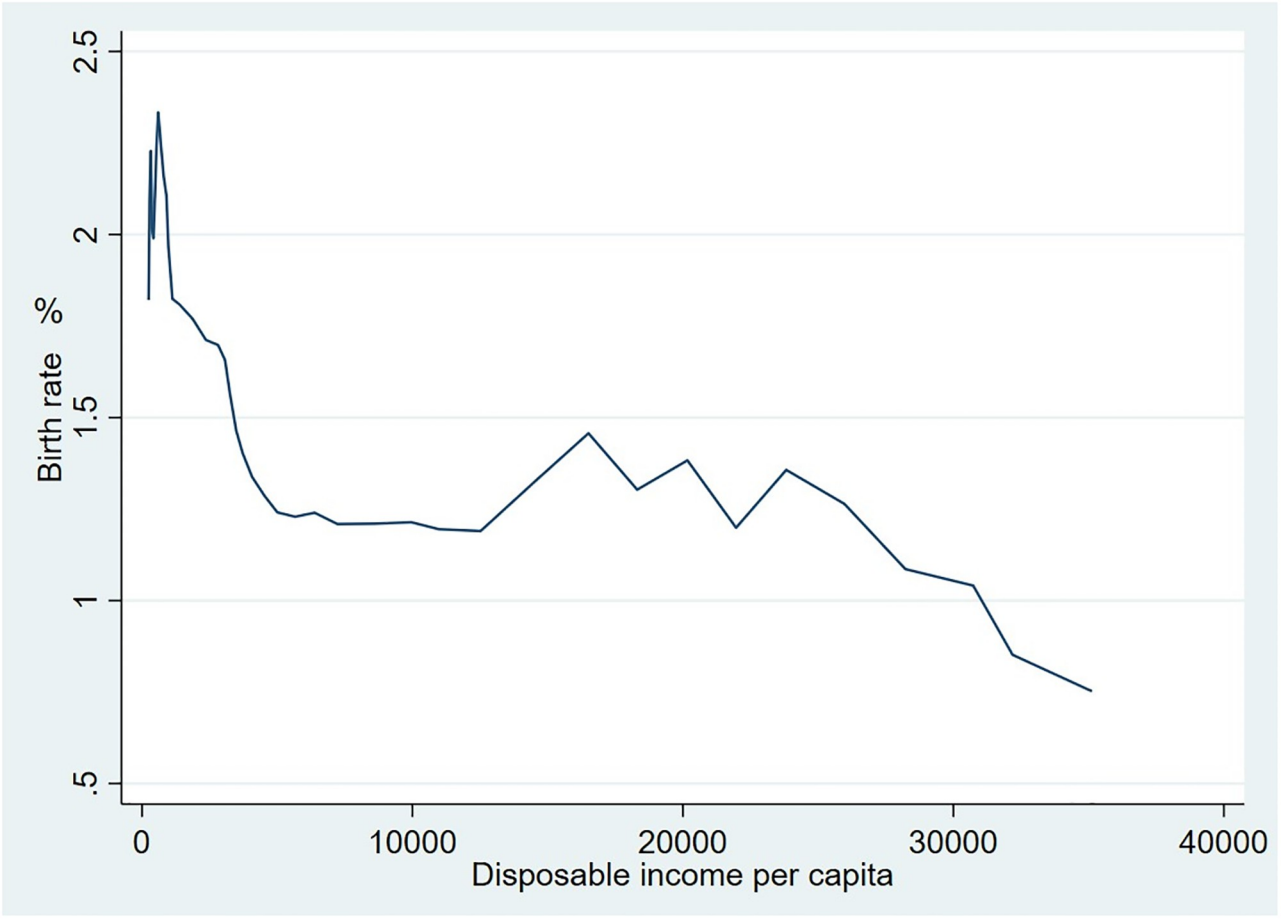
From 1980 to 2021 Relationship between birth rate and disposable income per capita.

To further study the relationship between birth rate and per capita disposable income, we have selected the 1980–2021 data from the National Bureau of Statistics, and used Stata 16.0 software to analyze them. According to our study, the birth rate slightly rose from 1.82% in 1980 to 2.33% in 1987 before falling sharply to 1.19% in 2010, with a drop of 48.93%. Since 2017, the birth rate kept decreasing and by the year of 2021 the rate was only 0.752%, much lower than the international average. According to Fig 3, the birth rate decreases first as per capita disposable income rises; when per capita disposable income increases to RMB 10,000, the birth rate increases along with the per capita disposable income increases; but when per capita disposable income rises to RMB 23,000, the birth rate begins to fall as per capita disposable income increases. Thus, the birth rate and disposable income per capita first appear as an inverse J-shape and then as an inverted J-shape. Furthermore, the birth rate will also be influenced by factors such as related policies, the pursuit of the quality of life, educational attainment and regional disparities.

## 3. Empirical analysis

### 3.1 Model construction

To study the relationship between birth rate and disposable income per capita, this paper firstly established a multivariate baseline regression model. It has set explained variables FET, explanatory variables PCDI, and control variables MR, FER, EL and BP. It has investigated the relationship between birth rate and disposable income per capita through descriptive statistics, correlation analysis and baseline regression analysis. Finally, the accuracy and reliability of the benchmark regression results are tested by collinearity test and robustness test, which further proved the validity of the results of the benchmark regression analysis. The benchmark regression model is constructed as follows.

$$FET_{it}=\beta_{0}+\beta_{1}PCDI+\beta_{2}PCDI^{2}+\sum \beta_{j}C_{it}+\gamma_{i}+\pi_{i}+\varepsilon_{it}$$

where, β represents the coefficient; FET denotes birth rate; PCDI is per capita disposable income; C_it_ means a series of control variables; γ_i_ stands for individual fixed effects; π_i_ is individual time effects, and ε_it_ represents random disturbance terms.

### 3.2 Variable selection and description

Based on the availability and representation of data, this paper selects data from 1980–2021 in China for the empirical analysis. The variables include 3 categories: explained variables, explanatory variables and control variables.

1. Explained variable: birth rate (FET). This paper uses the birth rate of Chinese population to represent the birth rate, and the data are obtained from the National Bureau of Statistics.
2. Explanatory variable: per capita disposable income (PCDI). From the relationship between birth rate and per capita disposable income (Fig 3), we can see that the birth rate and per capita disposable income follows an inversed J-shape pattern first before changing into an inversed J-shaped pattern. The model uses the data of disposable income per capita and the square of disposable income per capita, which are obtained from the National Bureau of Statistics.
3. Control variables
  (1) Marriage rate (MR): An increase in the marriage rate will, to a certain extent, promote the birth rate. Therefore, the marriage rate is selected as the control variable. The data are obtained from the World Bank and the China National Bureau of Statistics.
  (2) Female employment rate (FER): Higher the female employment rate will reduce the willingness of women to have children, and thus lower the birth rate. Many companies believe that if women get pregnancy and give birth to babies, their working productivity may decrease. So, they may refuse to hire women if they choose to have children. Losing employment becomes a hidden cost of childbirth for women. To secure their employment opportunities, women may choose to have children later, less or no children. Therefore, this study selects female employment rate as the control variable. The data are obtained from OECD database.
  (3) Educational attainment (EL): this study adopts the percentage of family members with college degree or above as the measure of educational attainment. According to the questionnaire survey, families with higher education attainment have lower fertility intention and the birth rate. Therefore, the educational attainment is selected as the control variable. Related data are obtained from the World Bank database.
  (4) Fertility intention (BP): fertility intention can significantly affect birth rate. we have collected relevant data through a questionnaire survey, in which 1 stands for families willing to have children, and 0 means those who are unwilling to have children.

### 3.3 Descriptive statistics

According to Table 2, the average birth rate is 1.56%, which is at a low level. The minimum value of birth rate in 2021 is 0.76%, which is far below the international alert line. As the birth rate declined significantly in recent years, it is an urgent task for the government to increase the rate. As for the per capita disposable income, the minimum value is RMB 247 and the maximum value is RMB 35,128. With the continuous development of China’s economy, residents’ per capita disposable income keeps increasing. From the results of control variables, there are significant differences in fertility intention, marriage rate, educational attainment and female employment rate, etc. Looking at the maximum and minimum values and combined values over the years, we can find that the educational attainment and female employment rate are two values that are keep rising. The standard deviation of female employment rate is 12.99, and the difference between the maximum value of 56.1 and the minimum value of 14.28 is huge. This means that female employment rate has increased significantly in recent years. Moreover, the marriage rate of China keeps decreasing, with the minimum value of 0.56%. From 2020 and 2021, the marriage rate even dropped below 60%. The mean value of fertility intention is 0.42, which is less than 0.5. The decreasing fertility intention suggests that less families are willing to have children.

**Table 2:** Descriptive statistics of variables.

Variables	Symbols	Unit	Observations(1980–2021)	Minimum value	Maximum value	Average value	Standard deviation
Birth rate	FET	%	42	0.75	2.33	1.56	0.42
Disposable income per capita	PCDI	hundred dollars	42	2.47	351.28	88.63	103.25
Fertility intention	BP	-	42	0	1	0.42	0.47
Marriage rate	MR	%	42	0.56	1.04	0.78	0.12
Education level	EL	%	42	0.11	3.25	1.24	1.04
Female Employment Rate	FER	%	42	14.28	56.1	32.45	12.99

### 3.4 Correlation analysis

Table 3 reports the Peaeson correlation coefficients of the main variables. Among them, PCDI, EL, FER and FET were negatively correlated, and BP, MR and FET were positively correlated, and the correlation coefficients passed the 5% or higher level test.

**Table 3:** Correlation analysis.

	FET	PCDI	BP	MR	EL	FER
FET	1					
PCDI	-0.76*	1				
BP	0.444*	-0.3294*	1			
MR	0.35*	-0.0958	0.2165	1		
EL	-0.8616*	0.9302*	-0.3353*	0.0599	1	
FER	-0.9046*	0.9299*	-0.3431*	-0.1478	0.9596*	1

Note:

*indicates more than 5% significant level

## 4. Analysis of the empirical results

### 4.1 Analysis of baseline regression results

This study selected the panel data of China from 1980–2021 and adopted stata17.0 software to estimate the benchmark regression model. Table 4 shows the regression results, where column (1) examined the relationship between birth rate and disposable income per capita with a coefficient of -0.0030924 and significant at the 1% level. This indicates that disposable income per capita is negatively related to birth rate. The coefficient of the squared per capita disposable income is 0.0000145, which is significant at the 1% level. This indicates that higher per capita disposable income will increase the birth rate to a certain extent, but the coefficient is small, indicating that the increase is not significant. In other words, there is an "inverse J" type relationship between per capita disposable income and birth rate. The inflection point of Fig 3 shows when the per capita disposable income exceeds RMB 23,800, the birth rate decreases again. This suggests that the relationship of per capita disposable income and birth rate follow an "inverse J" pattern. The coefficient of PCDI is still negative when the control variables are included in column (3) and the squared term of per capita disposable income is not included. Thus, the negative relationship between per capita disposable income and birth rate still holds after the inclusion of control variables. The coefficient of PCDI^2^ is 6.73e-06 when the squared per capita disposable income term and control variables are included in (4), and the results of (4) show that the per capita disposable income at the turning point is RMB 12,520 and RMB 20,167. They are within the range of the values in the sample. Therefore, the relationship between per capita disposable income and birth rate follows an "inverse J" and "inverted J" patterns.

**Table 4:** Baseline regression results.

	(1)	(2)	(3)	(4)
PCDI	-0.0030924*** (0.000)	-0.007411*** (0.000)	-0.003282*** (0.000)	
PCDI^2^		0.0000145*** (0.005)		6.73e-06*** (0.000)
MR			0.8611361*** (0.000)	1.049141*** (0.000)
FER			-0.0281758*** (0.000)	-0.022979*** (0.000)
EL			-0.2960053*** (0.000)	-0.216464*** (0.000)
BP			0.05505071* (0.055)	0.0681519** (0.02)
Constant term	1.8353	1.952915	1.841639	1.587795
Individual effects	Yes	Yes	Yes	Yes
Point-in-time effect	Yes	Yes	Yes	Yes

Note:

***, **, * indicate significant at the 1%, 5% and 10% levels, respectively

Table 4 shows the results of the control variables. The marriage rate is significantly positively correlated with the birth rate. A higher marriage rate will promote birth rate but higher female employment rate will decrease the birth rate. This is because giving birth to new babies will affect women’s employment and promotion opportunities. Reluctant to pay such a high cost of childbirth, women will choose to have fewer children. Moreover, the educational level is significantly and negatively correlated with the birth rate. Women with higher educational level tend to have fewer children. The main reason may be families with higher educational attainment will have less gender discrimination and will focus on the quality of children’s education, thus pushing up their costs of education for children. As a result, the birth rate is reduced. Fertility intention is significantly and positively correlated with birth rate. In other words, families with stronger fertility intentions are likely to have more children and vice versa. This study has standardized the baseline regression coefficients of equation (3), as can be shown in Table 4. After comparing the scores, we discovered that the marriage rate has a more significant role in promoting fertility, and for every 1% increase in the marriage rate, the birth rate will rise by 0.86%. The educational attainment and female employment rate have more significant role in reducing the birth rate. For every 1% increase in the education level, the birth rate will decrease by 0.296%, and for every 1% increase in the female employment rate, the birth rate will decrease by 0.028%.

### 4.2 Co-linearity test

The vif covariance test was performed on equations (3) and (4) of Table 4. The test results were 9.74 and 8.06, which were less than 10 but greater than 5. This suggests that there is a certain linearity problem between the variables. After further analysis, it was discovered that there is a certain linear relationship between educational attainment and female employment rate. Therefore, the variable EL was excluded in post-exclusion regression. The results were still significant at the 1% level significantly, as shown in Table 5. Equations (7) and (8) show that increasing disposable income per capita will promote the birth rate. This is consistent with the results of benchmark regression. The results of the cointegration test are 4.35 and 2.13, which are less than 5. Therefore the model is well constructed and does not have the problem of multiple cointegration.

**Table 5:** Regression results after excluding the variable EL.

	(5)	(6)	(7)	(8)
PCDI	-0.0030924*** (0.000)	-0.007411***(0.000)	-0.0022625*** (0.000)	
PCDI^2^		0.0000145*** (0.005)		5.00e-06*** (0.000)
MR			0.6416071*** (0.002)	0.8578473*** (0.000)
FER			-0.0437611*** (0.000)	-0.0368319*** (0.000)
BP			0.1146855*** (0.008)	0.11628*** (0.004)
Constant term	1.8353	1.952915	2.196141	1.909932
Individual effects	Yes	Yes	Yes	Yes
Point-in-time effect	Yes	Yes	Yes	Yes

Note:

***, **, * indicate significant at the 1%, 5% and 10% levels, respectively

### 4.3 Robustness test

#### 4.3.1 Robustness test of replacement variables

For the robustness test, we changed the explanatory variables. Fig 1 shows that the birth rate and the natural population growth rate follow the same trend, so we used the natural population growth rate instead of the birth rate to perform the stability test. Through the regression analysis, we find that the disposable income per capita is negatively correlated with the natural population growth rate, with a coefficient of -0.0032941 and a significant P value of 0.000. According to the results, after replacing the explanatory variables, the regression results are consistent with the baseline regression results, which are significant at the 1% level. Therefore, the regression results are valid and have passed the robustness test.

#### 4.3.2 Robustness test of explanatory variables taking logarithms

To further test the relationship between birth rate and disposable income per capita, we performed the regression analysis on the logarithm of the explanatory variable disposable income per capita. Table 6 shows the results: the birth rate is negatively correlated with the logarithm of per capita disposable income and positively correlated with the logarithm of squared per capita disposable income. The relationship between disposable income per capita and birth rate is valid and follows the patterns of "inversed J" and "inverted J".

**Table 6:** Regression results after taking logarithm of the explanatory variable disposable income per capita.

	(9)	(10)	(11)	(12)
Ln(PCDI)	-0.2487013 *** (0.000)		-0.2540608*** (0.003)	
Ln(PCDI^2^)		0.1243436*** (0.000)		0.1270392*** (0.003)
MR			0.8507945*** (0.000)	0.8509743*** (0.000)
FER			0.0027791 (0.774)	0.0027829 (0.773)
BP			0.1100655** (0.037)	0.1100878** (0.037)
Constant term	2.452382	2.452316	1.636358	1.636127
Individual effects	Yes	Yes	Yes	Yes
Point-in-time effect	Yes	Yes	Yes	Yes

Note:

***, **, * indicate significant at the 1%, 5% and 10% levels, respectively

## 5. Conclusion and insight

### 5.1 Conclusion

China’s current birth rate is low. Moreover, the imbalance of the economic development in China results in a huge gap in the birth rate between the east and west. The birth rate in the eastern region is lower than the west. Through the comparison of birth rates in different provinces, we found that the birth rate in Yunnan and Guizhou has been maintained at more than 1.2%, mainly because the local ethnic minorities account for a larger proportion of the population, and China has preferential policy for them. The birth rates in Beijing and Shanghai have been declining over the past 5 years, and the birth rates in 2021 were only 0.635% and 0.467%. As first-tier cities, Beijing and Shanghai have more developed economies. Young people in Beijing and Shanghai have higher educational attainment, and their living, housing, childcare costs are very high. Under such pressure, they are less likely to have many children. The three northeastern provinces, especially Heilongjiang province, of which the birth rate was only 0.359% in 2021, have much lower birth rates than the national average. Due to the outflow of population, the birth rates of this region stay at the bottom of the country. For the first-tier cities, China should introduce more family-friendly policies, such as subsidies, allowances, tax breaks, maternity insurance to reduce the cost of living in these regions. For the three northeastern provinces, the government need to prevent the outflow of population by increasing both the number of jobs and the productivity as well as incomes from employment of this regions.

According to the empirical analysis, the relationship between per capita disposable income and birth rate is first "inverse J" and then "inverted J", indicating that increasing per capita disposable income will have some effect on birth rates. Women’s employment rate and education level are negatively related to birth rates, but the marriage rate and fertility intention are positively correlated with birth rates. To increase birth rates, China should lower the living costs and increase per capita disposable income. Expenditures in housing, education and medical care make for a relatively substantial share of residential spending. Therefore, to reduce the living cost of residents, we should first focus on housing, education and medical costs and on top of that China should also boost the marriage rate and fertility intentions to improve the birth rate.

### 5.2 Recommendations

China should issue more policies and tax relief to increase birth rate. Our recommendations are as follows:

#### 5.2.1 Child allowance

Child allowances are a major incentive to encourage childbirth and solve families’ financial difficulties. In Russia, there is a policy that provides for a one-time financial allowance for each newborn baby, equivalent to 2.6 times the country’s average annual wage. In Germany, parental allowance is a minimum of 300 euros and a maximum of 1,800 euros monthly. In Japan, women being four months pregnant are entitled to a variety of benefits. To reduce the financial burden of childbirth for families, the government will also provide a subsidy of ¥100,000 for every newborn. Like other countries, China should also increase the types and amounts of allowances to complement maternity insurance. Moreover, the government should provide additional benefits to families or encourage companies to hire more women by providing incentives for such hiring.

#### 5.2.2 The pre-tax deduction for children’s education

As the economy develops and education levels increase, parents have higher expectations for their children and invest more in education. In Germany, parents can make a tax deduction on their income based on their children’s education; in the United States, some education expenses are tax deductible or may allow taxpayers to claim a tax credit. For French residents, the deduction varies according to the age and education stages of the child. China should increase the pre-tax deduction for children’s education. Currently, for children’s education, an amount of 1,000 yuan ($145) will be deducted every month from the parents’ taxable income for each child’s education. However, the educational costs are more expensive in China. Therefore, the government should consider to increase deduction amount to compensate the parents.

#### 5.2.3 Childcare and pre-tax deduction

The use of childcare has become increasingly common. Such care is essential for families where both parents work outside the home, and many other families choose to place their children in childcare in an effort to better prepare them for school. Many countries provide deductions associated with childcare. For example, in Germany, parents can deduct up to 4000€ in services related to childcare, when hiring nurses or going to a child care center, etc. The provision of childcare facilities in France relaxes the parents’ time constraints and alleviates the parental burden in terms of childcare. In China, a growing share of families are unwilling to give birth, because childbirth is expensive and they do not have the help from family relatives, particularly grandparents, to take care of their children. To address this issue, China should increase its child care capacity, while provide tax benefits to offset child care costs, such as the child care tax credit.

#### 5.2.4. Child medical expenses deduction

In recent years, families’ spending on children’s health care, from infancy to early childhood, has risen considerably. To ease the financial pressures for new parents, many governments have enacted health-care policies for children. In Germany, parents can make tax savings by deducting their expenses for all medical expenses of their children; Russia introduced income tax deductions for children’s medical expenses, including medical expenses for children under 18 years old, and life insurance or supplementary medical insurance premiums for children over 5 years old. Currently, Chines residents can only deduct the medical cost that is over RMB 15,000 and borne by the individuals, but such deduction does not cover the medical expenses of their children. To free parents from the financial cost of childbirth, the government needs to cover this part and increase the deduction for children’s medical expenses.

#### 5.2.5 Housing costs and educational expenses

Chinese residents are facing sky-high mortgage and housing prices, especially for the “the school district houses”. The policy of “nearby enrollment” was enacted to equalize the opportunities for students to enter schools, narrow the gap between key schools and weaker schools. However, most families want to invest more in each child to give them the best opportunities to compete in an increasingly unequal environment. The actual outcome of the mania for “school district house” was that the price of housing around better schools increased. China should reduce mortgage interest, offer housing and rental subsidies or tax incentives, and increase its investment in education. The government need to decouple property and education by delinking students’ educational options from their residential addresses, thus reducing housing and education costs.

#### 5.2.6 Increase the willingness to have children

At present, China’s marriage rate is reduced. Men and women with higher educational attainment tend to pursue better life quality. They set a high bar in the hopes of finding the perfect partner, resulting in a growing numbers of single population. In China, the legal age for marriage of men and women is 22 and 20 years old, respectively. In most developed countries, the legal age for marriage is 18 years old. Under special circumstances, it can be lowered to 16 years old. To increase the birth rate, China can lower the legal age of marriage. Meanwhile, it can lower the divorce rate by providing family therapy or extending the divorce cooling-off period. In addition, for couples who are willing to have children, they can be exempted from the marriage restriction and the society should provide parents basic childcare support and preferential policies, so that to increase fertility intentions and birth rates.

## Supporting information

S1 Data (XLS)
